# Neural network and regression approaches for predicting bubble point pressure in oil reservoirs

**DOI:** 10.1038/s41598-026-49027-8

**Published:** 2026-04-30

**Authors:** E. M. Mansour, Sayed Gomaa, A. N. El‑hoshoudy

**Affiliations:** 1 PVT Lab, Production Department, Egyptian Petroleum Research Institute, Cairo, 11727 Egypt; 2 PVT Service Centre, Egyptian Petroleum Research Institute, Cairo, 11727 Egypt; 3 Mining and Petroleum Engineering Department, Faculty of Engineering, Al- Azhar University, Cairo, Egypt

**Keywords:** Oil reservoirs, Bubble point pressure, Artificial neural network (ANN), Regression analysis, PVT fluid properties, Energy, Chemical engineering

## Abstract

Petroleum engineers must accurately determine bubble point pressure (Pb) to optimize oil field development from discovery to abandonment. Errors in predicting this parameter can lead to inaccurate reservoir and production estimates. To enhance Pb predictions, A total of experimentally measured 1,161 oil samples from Egyptian oil reservoirs (604 heavy oil and 557 light oil) were used for regression analysis. In contrast, 232 datasets were used to support the artificial neural network model. Existing correlations for Egyptian crude oils of varying oil gravity demonstrated moderate accuracy, highlighting the need for improved estimation methods tailored to local reservoir conditions. This study revises existing literature correlations, which previously related Pb to reservoir temperature, flashed gas specific gravity, gas-oil ratio, and stock tank oil API gravity. In contrast, this work develops two new correlation models: one tailored for heavy oils (15.3 < API < 34.9) and another for light oils (35.6 < API < 53.1), providing improved accuracy for these distinct oil types. These models incorporate a compositional approach, emphasizing fluid composition and physical parameters for enhanced precision. The equations were revised using multiple linear regression analysis, resulting in a statistical coefficient of 99.08% for heavy oils and 96.49% for light oils. Additionally, Artificial Neural Network (ANN) models were trained on a comprehensive dataset to develop highly accurate mathematical models for predicting bubble point pressure.

**Supplementary Information:**

The online version contains supplementary material available at 10.1038/s41598-026-49027-8.

## Introduction

**Fig. 1:**
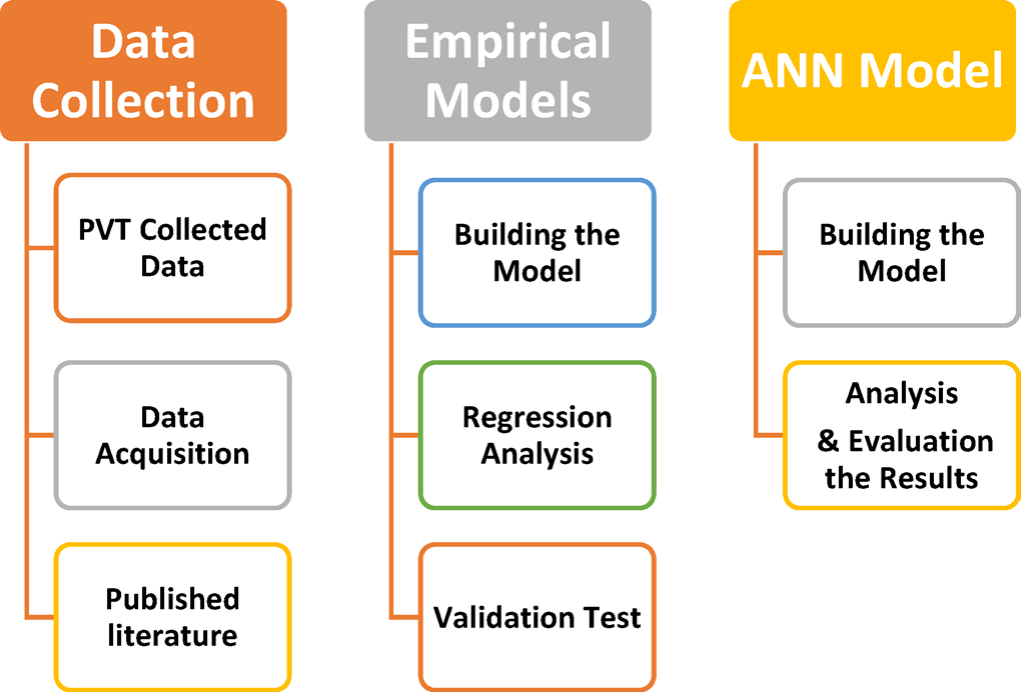
The framework of research methodology.

Bubble Point Pressure is a vital primary property in Pressure-Volume-Temperature (PVT) analysis, serving as a cornerstone for calculations in reservoir and production engineering. It is essential for reservoir management, pipelines and facilities design, petroleum production, and material balance^1^. This parameter can be directly detected in experimental PVT cells, but this process is more time-consuming and costly. Throughout the previous seven decades, Most researchers have developed various empirical correlations tailored to specific geological regions due to variations in the composition of reservoir fluid^2–4^. Most Pb correlations rely on linear, non-linear, or multiple regression techniques. They are primarily influenced by key factors such as reservoir temperature, gas-oil ratio, API gravity, and gas composition^5^. Al-Marhoun (1988)^6^ developed a correlation for estimating Pb based on 160 experimental data points sourced from 69 oil reservoirs in the Middle East. for Pb calculations, the correlation’s average absolute relative error was 3.66%. Labedi (1990)^7^ conducted a study using laboratory measurement samples from three major oil producers focused on African crude oil systems. The study included 97 samples from Libya, four from Angola, and 27 from Nigeria. Labedi estimated a correlation to estimate Pb based on critical parameters, including stock-tank oil gravity, solution gas/oil ratio, reservoir temperature, and gas gravity. Dokla and Osman (1992)^8^ introduced a novel correlation to determine Pb, tailored specifically for crude oils from the United Arab Emirates. Using 51 data sets, they recalibrated the coefficients of the existing Middle East correlations developed by Al-Marhoun 1988. Their correlation achieved an average error of just 0.45% for Pb estimation. Using 90 data sets from the Gulf of Mexico, Petrosky Jr. and Farshad (1993)^9^ created a new correlation for estimating Pb. They enhanced the adaptability of preexisting correlations to better fit the dataset by utilizing nonlinear regression techniques. De Ghetto, Paone, and colleagues (1994)^10^ used a dataset of 195 samples gathered from the North Sea, the Persian Gulf, Africa, and the Mediterranean Basin to establish a new correlation for predicting Pb. When compared to Pbcorrelations already found in the literature, their investigation, which included over 3,700 data points, showed errors of less than 10%. Hanafy, Macary et al. (1997)^11^ introduced a new correlation for estimating Pb depending on PVT measurements from 324 data sets gathered from 75 oil fields across three distinct regions in Egypt. Covering a spectrum of oil types from heavy to volatile oils, this correlation offered a more precise approximation of Pb specifically for crude oils in Egyptian fields. Al-Shammasi (1999)^12^ determined a numerical correlation for estimating Pb using a comprehensive global data bank comprising 1,243 measurements from various locations worldwide. Mehran, Movagharnejad et al. (2006)^13^ developed a nonlinear multiple regression model to predict reservoir Pb using 387 published data collected from Iranian crude oils. The model achieved an absolute average per cent error of 9.3%, a standard deviation of 28.3%, and a correlation coefficient of 0.895. The results indicated that this new model provided more accurate predictions compared to previously established empirical correlations. Mazandarani and Asghari (2007)^14^ conducted PVT analysis on 55 samples from various Iranian oil fields to create a correlation for predicting Pb. Their model was based on Al-Marhoun’s correlation and utilised linear regression analysis performed with Eviews software. The derived correlation expressed Pb as a direct function of oil gravity, reservoir temperature, gas-to-oil ratio, and gas gravity. Emara (2015)^15^ created a correlation to estimate the Pb of Egyptian crude oils using nonlinear multiple regression. The correlation was calculated using 178 data points taken from several oil fields in Egypt and expressed as a function of reservoir temperature, oil API gravity, gas relative density, and solution gas-oil ratio. Ikpabi, Akinsete et al. (2022)^16^ present a machine learning-based model for predicting Pb using 314 PVT data points from the Niger Delta. By linearizing nonlinear regression and applying white-box models, the Linear Regression approach proved most accurate, highlighting the efficiency of machine learning when experimental data is unavailable. Arwini (2024)^17^ improves the Al-Marhoun correlation for estimating Pb in black oil reservoirs using linearized non-linear regression, specifically for Libyan crude oils. Using 62PVT datasets, the enhanced model achieved high accuracy, with R² = 96.70%, APE = 0.70%, and SD = 14.70, outperforming the original correlation. This novel correlation fared better than previous published Pb correlations, according to statistical and graphical analyses^15^. Artificial neural networks (ANNs) are increasingly important in predicting physical properties in petroleum engineering due to their ability to model complex, non-linear relationships between input variables and output properties^18^. In petroleum reservoirs, physical properties such as Pb, viscosity, and porosity can be influenced by numerous factors, often making traditional methods of prediction cumbersome and less accurate. ANNs, with their ability to learn from vast amounts of data, offer a powerful tool to capture intricate patterns in reservoir characteristics and fluid behavior^19^. By training on historical data, ANNs can provide fast, reliable predictions that improve decision-making in exploration, reservoir management, and production optimization, ultimately leading to more efficient and cost-effective operations in the petroleum industry, as shown in supplementary material (Table S1)^19,20^. Several models have been created to estimate Pb using an ANN. These models employ input data such as reservoir temperature, gas-specific gravity, gas-oil ratio, and API gravity. Moghadam, Salahshoor et al. (2011)^21^ utilised the ANN technique to predict the Pb of Iranian crude oils. Their model achieved a high accuracy with a correlation coefficient of 0.990, demonstrating the effectiveness of ANN in Pb estimation. Al-Marhoun and Osman (2002)^22^ created a new model for determining Pb using an ANN and 283 data points collected from various locations in Saudi Arabia. The model achieved an average absolute error of 5.8% and outperformed all previously established equations for Pb estimation. El-Sebakhy, Sheltami et al. (2007)^23^ employed the Support Vector Machine (SVM) framework to develop a model for determining Pb. Their findings revealed that the SVM model outperformed traditional empirical correlations in addition to ANN models in terms of accuracy and reliability. Moghadassi, Parvizian et al. (2009)^24^ emphasised that the ANN model is one of the most successful for forecasting Pb. They optimised the hidden layer by configuring it with sixty neurons, resulting in a minimum Mean Squared Error (MSE) of 0.000606. Numbere, Azuibuike et al. (2013)^25^ applied an ANN method in the Niger Delta to develop an empirical equation for Pb, utilising 1,248 data points. The data was separated into three sets: training (60%), validation (20%), and testing (20%). Their ANN model outperforms existing empirical correlations for the region. Baarimah, Gawish et al. (2015)^26^ estimated Pb using a Fuzzy Logic (FL) model, achieving a correlation coefficient of 0.9995. The model incorporated inputs such as gas-specific gravity, dissolved gas-oil ratio, oil-specific gravity, and reservoir temperature. Adeeyo (2016)^27^ used a neural network to estimate the Pb of crude oil in Nigeria, employing 2,114 data points. He tested various neural network architectures and neuron configurations, dividing the data into 60% for training, 20% for validation, and 20% for testing. As a result, his model’s accuracy surpassed that of already published models. Using Artificial Intelligence (AI) approaches, Elkatatny and Mahmoud (2018)^28^ predicted bubble-point pressure Pb based on dissolved gas-to-oil ratio, oil specific gravity, reservoirtemperature, and gas specific gravity. Their model achieved a high correlation coefficient of 0.988 and an average absolute error of 7.5%. Ghorbani, Wood et al. (2020)^29^ proposed four models for accurately estimating Pb in the Ahvaz oil field in Iran. Among them, the CHPSO-ANFIS model performed the best, delivering a correlation coefficient of 0.9992, a root mean square error of 43.21, a standard deviation of 0.0126, and an average absolute relative deviation of 0.846, producing highly acceptable outputs. Alakbari, Mohyaldinn et al. (2022)^30,31^ developed and validated a model using the adaptive Neuro-Fuzzy Inference System (ANFIS) to accurately and reliably forecast Pb based on over 700 global datasets. The model achieved excellent performance with the lowest average absolute percentage relative error (AAPRE) of 6.38%, average percentage relative error (APRE) of −0.99%, standard deviation (SD) of 0.074, and root mean square error (RMSE). Additionally, it attained a maximum correlation coefficient (r) of 0.994. Most of the published correlations were developed using a single equation to calculate Pb for specific locations with a broad range of oil-specific gravities. However, these models cannot be generalized to accurately predict Pb properties across diverse geological settings with varying oil characteristics^3,32^. The study framework, as shown in Fig. 1, encompasses data collection and the development of empirical correlations, as well as mathematical models, to address the significant gap in accurately predicting Pb using existing correlations for black and volatile oil reservoirs. This is achieved by introducing two novel empirical correlations for these types of reservoirs and employing ANN models based on various API gravities and physical parameters to estimate bubble point pressure accurately.

## Methodology & data acquisition

**Fig. 2:**
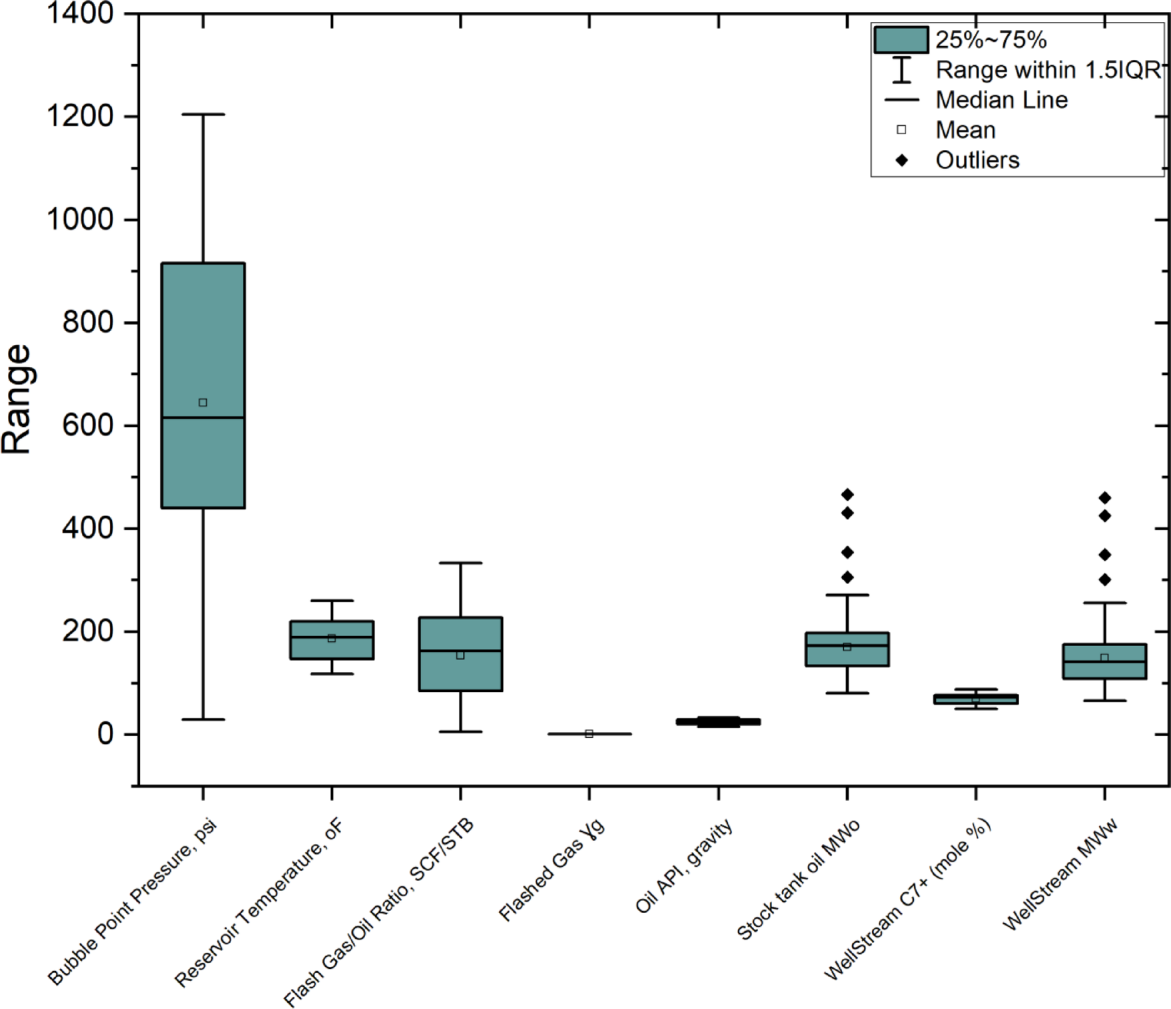
Box Plot of the used variables in correlation development.

In this study, data from various oil fields were collected, and 1,161 laboratory PVT analyses were conducted. The Pb was measured in the laboratory using a constant mass depletion (CMD) test within a PVT cell. In the PVT cell, a reservoir fluid sample was exposed to both an excess starting pressure and the reservoir temperature^33^. The pressure-volume relationships of the sample were used to determine the Pb and the corresponding volume. Additional physical properties were determined through primary laboratory studies, including flashing tests and compositional analyses^34,35^. The dataset includes parameters such as reservoir temperature, heptane-plus content of the well stream, flashed gas specific gravity, Pb, molecular weight of stock tank oil (STO), well stream, solution gas-oil ratio, and oil API gravity. These physical properties parameters fall within the ranges presented in Table 1; Fig. 2. The collected data were obtained entirely from our experiments. They were classified into two groups: the first group comprised 604 datasets for heavy oil, with API gravity ranging from 15.3 to 34.9, while the second group included 557 datasets for light oil, with API gravity ranging from 35.6 to 53.1. These experimental datasets were used for regression analysis. Additionally, A total of 232 datasets were used for the development and validation of the ANN model.

**Table 1:** PVT Collected Data.

Input Parameters	Minimum	Maximum	Mean	Median
In the case of heavy oil (15.3 < API < 34.9)				
Reservoir temperature **(**T_res_**)**, ^°F^	104	260	186.99	187.1
Gas oil ratio (R_s_), SCF/stb	5.4	333.0	141.19	142.9
Bubble point pressure **(**Pb**)**, psi	20	1204.6	602.30	611.1
Oil gravity (API)	15.3	34.9	25.02	25.2
Flashed gas gravity (**γ**_**g**_**)**	0.6915	0.8974	0.77	0.8
The molecular weight of oil (MW_o_)	122.2	207.4	178.00	176.7
Heptan plus of well stream (C_7_ ^+^ _W.S_)	50.4	89.9	69.96	72.0
The molecular weight of the well stream (MW_W.S_)	106.9	184.5	157.12	151.8
In the case of light oil (35.6 < API < 53.1)				
Reservoir temperature (Tres), °F	137.0	304.4	204.11	200.9
Gas oil ratio (Rs), SCF/stb	107.6	1780.3	599.86	526.2
Bubble point pressure (Pb), psi	290	4500	1594.21	1532.3
Oil gravity (API)	35.6	53.1	41.57	39.5
Flashed gas gravity (γg)	0.6215	0.8347	0.7375	0.7305
The molecular weight of oil (MWo)	125.0	200.5	154.61	152.6
Heptan plus of well stream (C7 + W.S)	19.7	76.6	46.87	46.5
The molecular weight of the well stream (MWW.S)	61.3	157.8	104.99	100.8

**Fig. 3:**
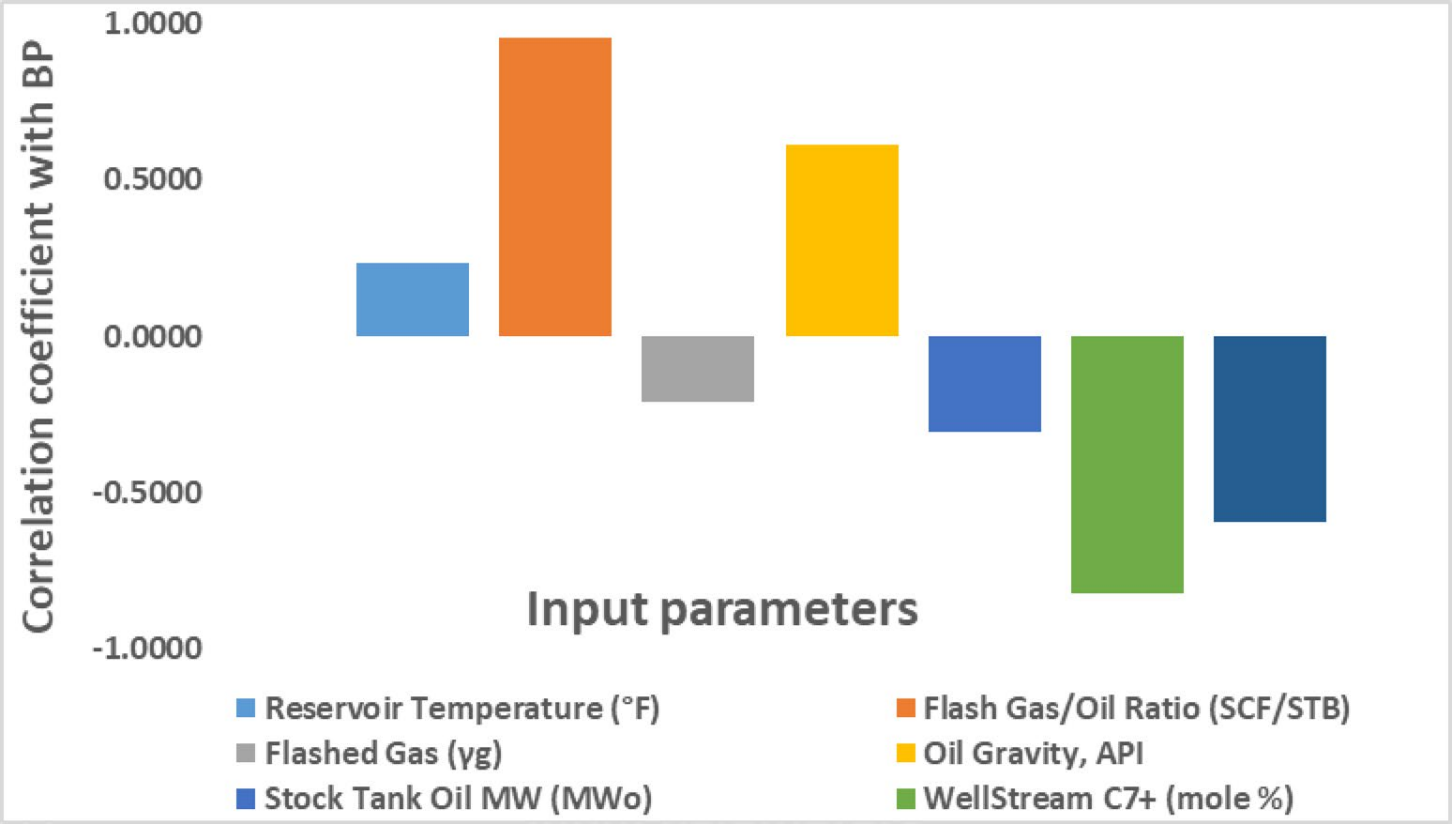
Effect of input parameters on bubble point pressure.

Before establishing the correlation, we should assess the influence of input physical properties parameters on the Pb. The impact of each variable on the prediction tends to decrease as the percentage values of the specified independent variables decrease^32,36^. Additionally, the relative effect of the variables in the trained set may vary across different datasets^37,38^. The impact of input physical properties parameters on Pb is seen in Fig. 3. The Pb is strongly positively correlated with oil API gravity and the solution gas-oil ratio, with coefficient determinations of 95.14% and 61.54%, respectively. In contrast, the Pb is inversely related to reservoir temperature, heptane-plus content in the well stream, flashed gas specific gravity, molecular weight of the oil, and molecular weight of the well stream, with coefficients of −23.22%, −81.82%, −21.00%, −30.45%, and − 59.32%, respectively. These relationships were evaluated using the correlation coefficient, which quantifies the strength and direction of the linear relationship between variables, and is calculated according to the supplementary material (Table S2).

## Results and discussions

**Fig. 4:**
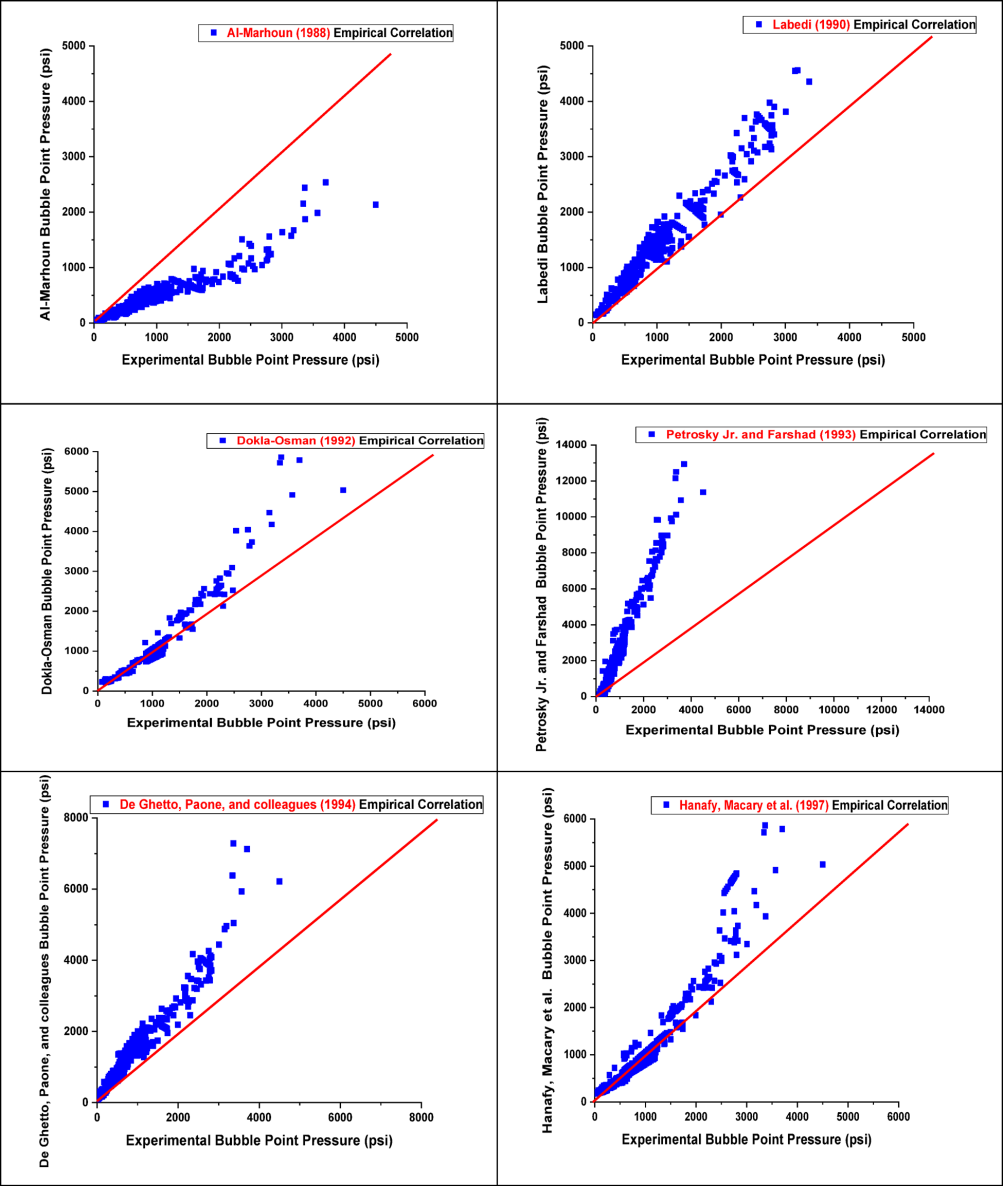

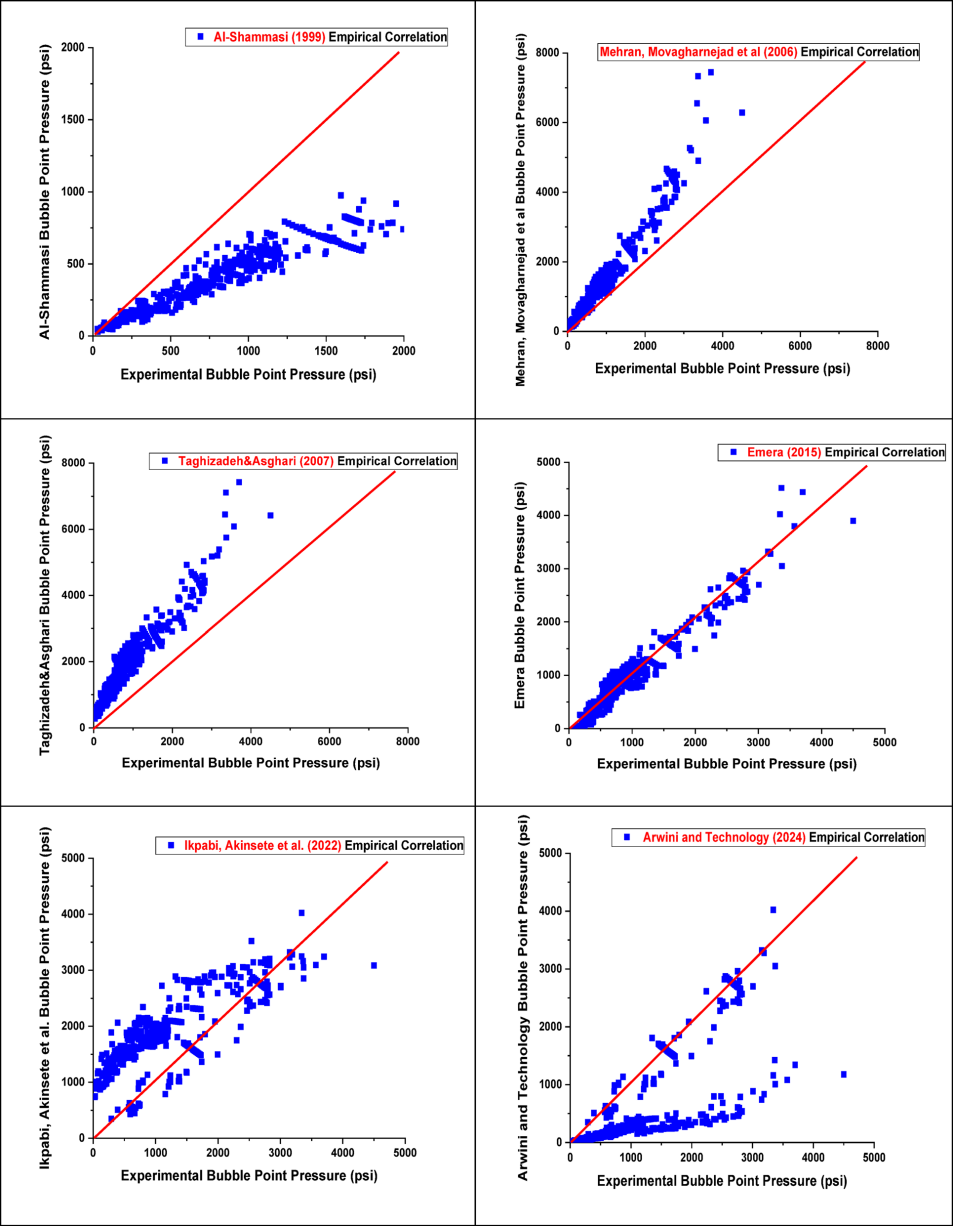
Cross plots of Pb for published empirical correlations.

Most existing PVT correlations, designed to estimate reservoir oil characteristics at reservoir temperature, are necessary for building equipment for surface operations and assessing the performance of reservoir inflow^39,40^. However, applying these correlations can be challenging without PVT analysis, as they often require parameters as input data, such as specific gas gravity and GOR. In many cases, the only necessary input data are separator GOR, separator pressure, stock-tank oil gravity, and reservoir temperature, which are rarely monitored in the field^41^. The accuracy of the published correlation is tested against the current data to validate its accuracy in predicting the Pb. The results of applying these literature correlations compared to field data from different reservoirs of black and light crude oil, taking their limitations into account, were evaluated to determine how the correlation outcomes aligned with the experimental values. Finally, we employed a statistical method to assess the relationship between the output Pb and the input data (GOR, reservoir temperature, and gas gravity). To quantitatively evaluate the model’s predictive performance, several performance evaluation metrics were used, such as relative error (E_r_), absolute error (E_a_), standard deviation (SD), correlation coefficient (r), maximum absolute error (E_max_), and minimum absolute error (E_min_), where all this abbreviations and symbols are clearly defined in supplementary material (Table S3). These metrics provide insight into the accuracy, consistency, and robustness of the developed model. The mathematical formulations and definitions of these metrics are presented in the supplementary material table(S2)^42^. The best literature correlation was selected based on the highest correlation coefficient and the lowest average absolute error percentage, as shown in Table 2 and supported by graphical analysis in Fig. 4. From the results in Table 2; Fig. 4, we concluded that the most accurate correlation for estimating Pb is Petrosky Jr and Farshad (1993)^9^ & Al-Shammasi (1999)^43^, as it relies heavily on reservoir API oil gravity, gas gravity, and temperature. This correlation demonstrated the highest performance with *r* = 0.9671 and 0.9291, as well as the lowest average absolute error percentage (13.94) for Dokla-Osman (1992)^8^. In another case, the poorest performance was observed in the correlations developed by Mehran, Movagharnejad et al. (2006) and Mazandarani & Asghari (2007), which yielded very low *r* = 0.039 and 0.042, respectively. These correlations were originally derived from datasets specific to geographical formations, which differ significantly from Egyptian oils in terms of fluid composition, API gravity, gas-oil ratio, and other critical reservoir properties. The poor performance can be attributed to regional and geological differences, including basin type, depositional environment, and thermal maturity. These factors have a significant impact on hydrocarbon generation, migration patterns, and overall fluid behavior, leading to poor correlation performance when applied without appropriate regional calibration^42^. Similarly, poor results were also obtained from the correlations developed by Ikpabi, Akinsete et al. (2022), and Arwini and Technology (2024), which further confirms the impact of regional and geological variations on the predictive accuracy of such models.

**Table 2:** Statistical error analysis of published empirical correlations.

Correlations	E_*r*_	E_a_	S_D_	*r*	E_max_	E_min_
^6^Al-Marhoun (1988)	47.29	47.58	12.25	0.4800	68.63	0.1636
^7^ Labedi (1990)	−41.99	42.02	22.17	0.7086	177.75	0.0507
^8^Dokla and Osman (1992)	−8.91	13.94	20.97	0.8691	182.75	0.1121
^9^Petrosky Jr. and Farshad (1993)	−56.85	213.76	339.57	0.9671	3192.28	0.3132
^10^De Ghetto, Paone, and colleagues (1994)	−74.56	74.79	46.19	0.4727	626.75	6.9017
^11^Hanafy, Macary et al. (1997)	−25.28	31.44	64.43	0.3617	816.98	0.1121
^12^Al-Shammasi (1999)	−57.36	57.56	49.82	0.9291	580.59	0.5377
^13^Mehran, Movagharnejad et al. (2006)	−67.44	67.67	43.83	0.0397	553.55	1.7219
^14^Mazandarani and Asghari (2007)	−173.26	173.26	143.26	0.0425	1838.41	31.0544
^15^Emara (2015)	29.35	46.89	114.61	0.7425	1205.41	0.0058
^16^ Ikpabi, Akinsete et al. (2022)	−205.04	205.34	266.96	0.1237	2441.76	0.2697
^17^ Arwini and Technology (2024)	75.04	75.04	6.10	0.2285	86.76	34.9617

### Building a new empirical correlations model using regression analysis

**Fig. 5:**
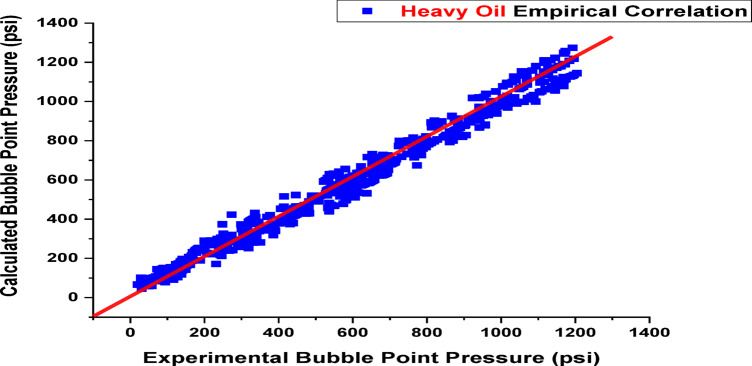
Cross plot of Pb for heavy oil (15.3 < API˚<34.9) [The current study].

**Fig. 6:**
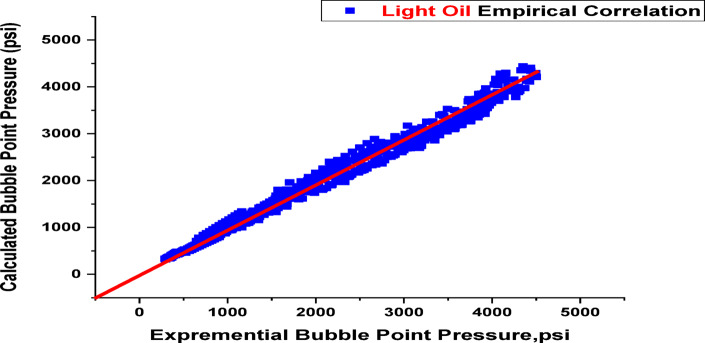
Cross plot of Pb for light oil (35.6 < API < 53.1) [The current study].

When developing a new model with a dataset, it’s essential to thoroughly analyze the data for potential errors, such as issues in data collection, encoding mistakes, or flaws in data selection and analysis methods^33,44^. The results and validation of the new model’s correlations are shown in this section, along with a comparison with correlations found in the literature, as seen in Table 2. Since then, the documented empirical correlations for bubble points have evolved in connection to the temperature of the reservoir, the specific gravity of flashing gas, the gas oil ratio, and the API gravity of stock tank oil^45,46^. This study develops two new empirical correlations: one for heavy oils with a range (15.3 < API < 34.9) and another for a range of light oils (35.6 < API < 53.1), This is impacted by the compositional model, which guarantees extremely accurate findings because it is more exacting and based on physical processes, composition of the fluid and, such as heptane plus (C_7_^+^) of well stream, molecular weight of oil, and well stream. This comparison represents the statistical error in the literature’s original correlations and those of the new models, which are comparable according to the lowest E_a_ and the highest r. Equation 1 illustrates how multiple-line regression analysis was used to create the suggested relationships. To ensure their accuracy, both quantitative and graphical residual analyses were conducted. The newly proposed Pb correlations offer more improvement and greater accuracy than those found in the literature, as shown in Table 3. Prediction results with an average per cent relative error of −6.44%, an absolute average per cent relative error of 11.90%, a standard deviation of 22.85%, and a correlation coefficient of 99.08% are provided by the recently proposed Pb correlation for heavy oils of range 15.3 < API˚<34.9. Also, prediction results with an average percent relative error of 8.02%, an absolute average percent relative error of 18.19%, a standard deviation of 24.35%, and a correlation coefficient of 96.49% are provided by the suggested Pb correlation for light oils (35.6 < API < 53.1). As seen in Figs. 5 and 6, both models well describe the data, as evidenced by the normally distributed residuals of both correlations. The suggested two models of bubble point correlations were compared to the earlier studies published bubble point correlations, and good results were obtained. The proposed models require data more closely tied to the specific oil samples, such as oil and gas molar composition.

**Table 3:** Statistical error analysis of the current study.

Correlations	E_*r*_	E_a_	S_D_	*r*	E_max_	E_min_
This study (Heavy oil)	−6.44	11.90	22.85	0.9908	250.57	0.0832
This study (Light oil)	8.02	18.19	24.35	0.9649	74.75	0.2782

1 $${\mathrm{Pb}}\,=\,\beta 0\,+{\beta _1}{{\mathrm{T}}_{{\mathrm{res}}}}\,+\,{\beta _2}{\mathrm{GOR}}\,+\,{\beta _3}{\gamma _g}\,+\,{\beta _4}{\mathrm{API}}\,+\,{\beta _5}{\mathrm{M}}{{\mathrm{W}}_o}\,+\,{\beta _6}{{\mathrm{C}}_7}{^{{\,+\,}}_{{\mathrm{W}}{\mathrm{.S}}}}\,+\,{\beta _7}{\mathrm{M}}{{\mathrm{W}}_{{\mathrm{W}}{\mathrm{.S}}}}$$

In the case of heavy oils **(15.3 < API˚<34.9)**, the constants are:

β_0_= −8.27 & β_1_ = 0.7538 & β_2_ = 2.4851.

β_3_ = 58.3 & β_4_= −1.991 & β_5_ = 9.176.

β_6_= −1.533 & β _7_= −8.901.

In the case of light oils **(35.6 < API < 53.1)**, the constants are:

β_0_ = 4727 & β_1_ = 2.377 & β_2_ = 0.591.

β_3_ = 5004 & β_4_= −3.71 & β_5_ = 21.94.

β_6_ = 13.46 & β _7_= −39.37.

### Validation of the new empirical correlations model

**Fig. 7:**
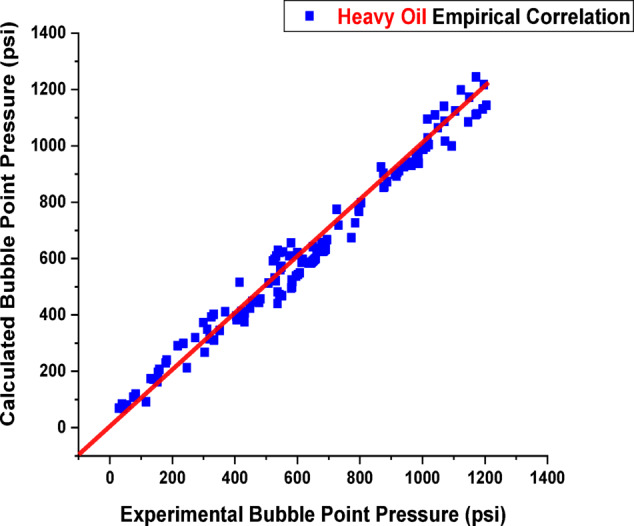
Validation of the developed correlation in the case of heavy oil.

**Fig. 8:**
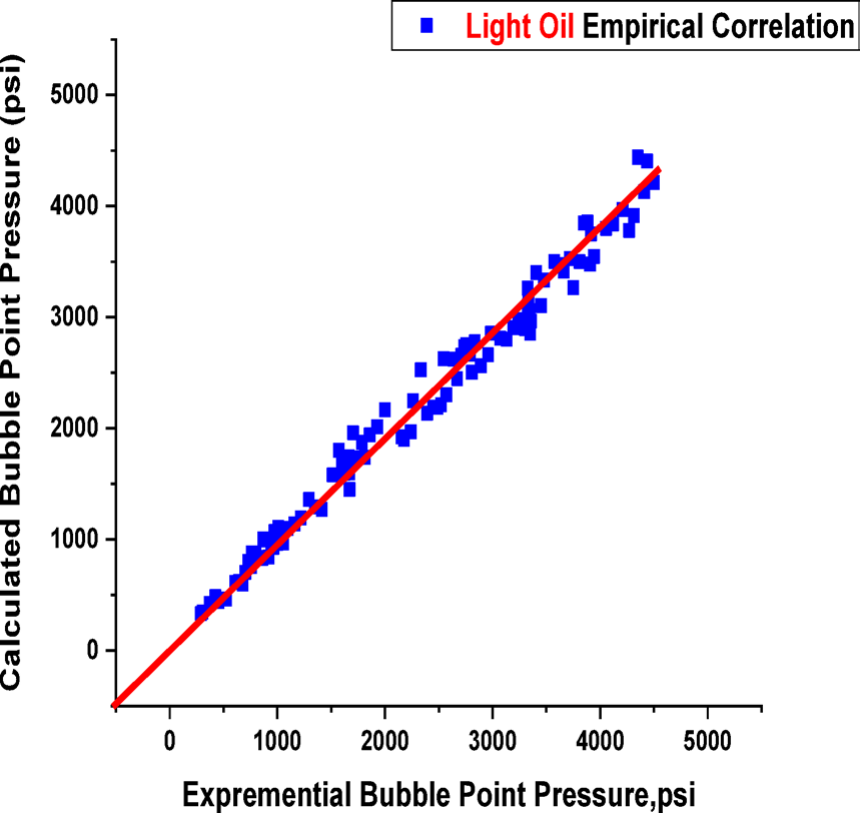
Validation of the developed correlation in the case of light oil.

In this section, the reported empirical correlations were tested for validity using selected (240) data sets, which were divided into two groups: (132) data sets used for heavy oil and (108) data sets in the second group used for light oil, which were not used during the correlation development process and all located within the range of the new correlations. Statistical and graphical error analysis were used to verify the validity. Validation of the newly developed correlations in the case of heavy and light oil is shown in Figs. 7 and 8, with a correlation coefficient of 0.9918 and 0.9832, respectively. Furthermore, Table 4 lists the details of the statistical error analyses for both heavy and light oil samples, which show a low relative error percentage and standard deviations. A comparison of empirical correlations between the Pb values from validation and the development one from the experimental data gives good agreement between the prediction and the tested data.

**Table 4:** Validation analysis of the developed correlation.

Correlations	E_*r*_	E_a_	S_D_	*r*	E_max_	E_min_
This study (Heavy oil)	−3.97	11.26	20.61	0.9918	138.05	0.6206
This study (Light oil)	4.26	17.53	15.31	0.9832	57.98	0.3746

### Development of an ANN model for predicting the bubble point pressure

**Fig. 9:**
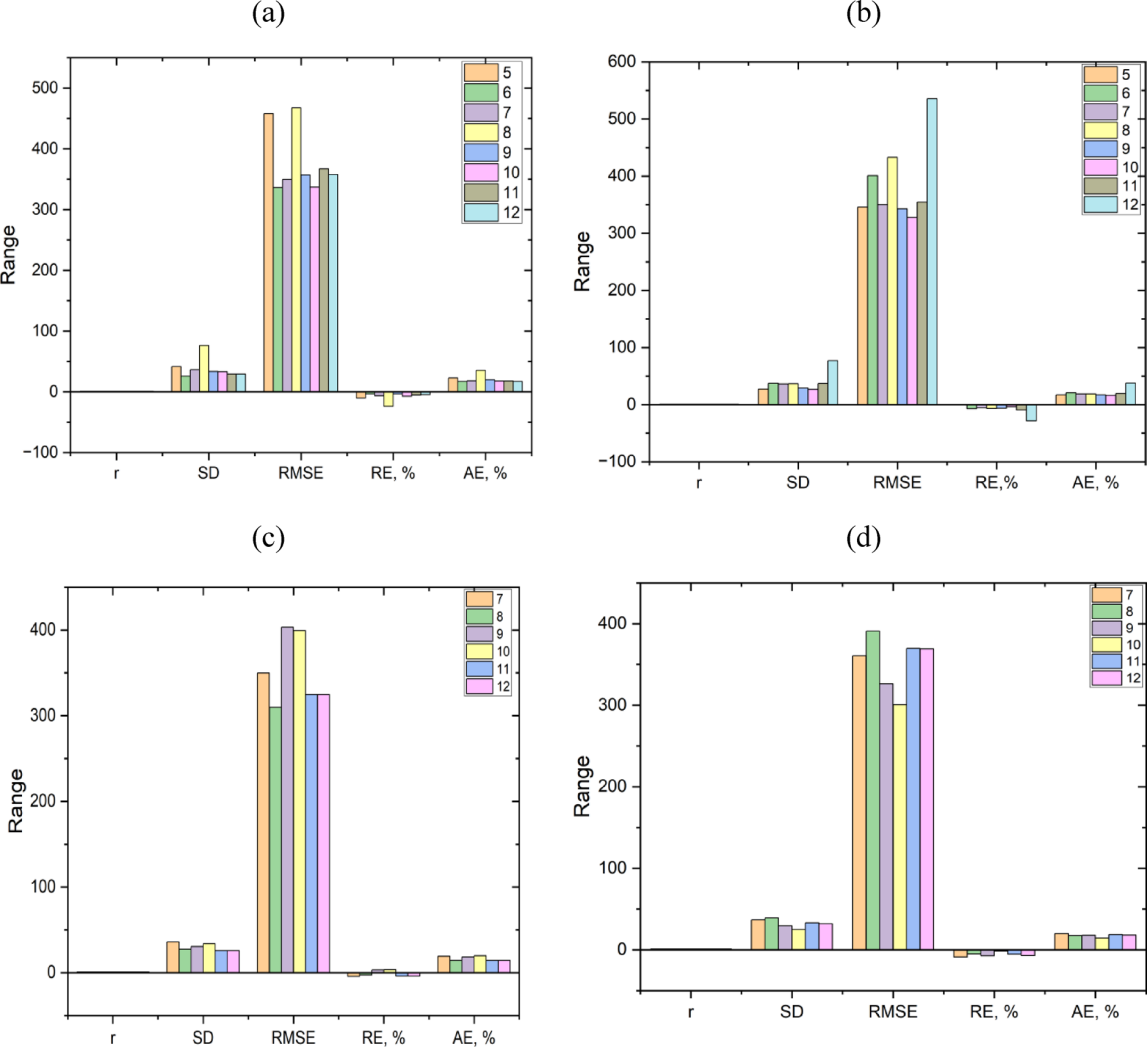
Visual representation of statistical parameters used for testing neurons’ accuracy at various numbers of neurons as follows: **a** in the hidden layer with Tansigmoid transfer function; **b** in the hidden layer with Logsigmoid transfer function; **c** in two hidden layers with Tansigmoid transfer function; **d** in two hidden layers with Logsigmoid transfer function.

The collected data sets are divided into three sets before building the ANN model to predict the bubble point pressure. A total of 232 datasets were divided into two sets: 70% (163 datasets) were used for training the model, and 30% (69 datasets) were allocated for validation and performance testing. Additionally, in order to construct the ANN model, all of the data are normalized between − 1 and 1^36,47^. To estimate the Pb as a function of solution GOR, gas density, oil density, and reservoir temperature, an Artificial Neural Network ANN model was created. Four layers form the foundation of the model. Four neurons serve as inputs in the input layer, which is the first layer. Two hidden layers include 10 neurons each. The fourth layer is the output layer, which has one neuron to predict the Pb output parameter. Firstly, the Tan-sigmoid and Logsigmoid are examined as transfer functions for a one-hidden-layer-based model with different hidden neurons (5, 6, 7, 8, 9,10,11,12) as presented in Tables 5 and 6, respectively. The optimum transfer function is Log-sigmoid with 10 neurons, where the coefficient of determination is 0.924 with an AARE of 0.16. Then, the effect of adding another hidden layer with various numbers of hidden neurons (7, 8, 9, 10, 11, and 12) on the model performance was reinvestigated, as presented in Tables 7 and 8. Furthermore, the visual representation of Tables 5, 6, 7 and 8 is illustrated in Fig. 9. The model performance is more accurate with two hidden layers and a log sigmoid transfer function. The pure-linear was investigated as the output function, and the Levenberg-Marquardt approach was chosen as the training procedure. Table 9 displays the attributes of the suggested model.

**Table 5:** Evaluation of ANN Pb model accuracy at various numbers of neurons in the hidden layer with Tansigmoid transfer function.

	5	6	7	8	9	10	11	12
*r*	0.836504	0.922067	0.910432	0.799115	0.909014	0.915324	0.908745	0.909925
SD	41.55538	25.84754	36.59998	76.1386	33.60764	33.32468	29.12717	29.04882
RMSE	457.9835	336.2552	349.5664	467.2029	357.1096	336.8318	366.8966	357.7551
RE, %	−10.372	−3.71117	−6.51979	−23.9671	−3.43655	−7.31487	−5.53717	−4.5212
AE, %	22.97302	16.83289	18.15968	35.47353	19.83411	17.65576	17.9126	17.07995

**Table 6:** Evaluation of ANN Pb model accuracy at various numbers of neurons in the hidden layer with Logsigmoid transfer function.

	5	6	7	8	9	10	11	12
*r*	0.918896	0.891634	0.912028	0.867718	0.915532	0.923678	0.908554	0.787904
SD	27.02798	37.40029	36.04126	36.34403	28.99354	26.64344	36.86399	76.71942
RMSE	345.6082	400.8912	349.9193	432.7524	342.7434	327.6705	354.2929	535.3165
RE,%	0.310394	−7.12531	−5.33095	−6.7741	−6.18845	−3.46414	−9.53269	−28.6066
AE, %	17.03247	20.60161	18.66645	18.49074	17.02793	16.29323	19.64564	37.68083

**Table 7:** Evaluation of ANN Pb model accuracy at various numbers of neurons in two hidden layers with Tansigmoid transfer function.

	7	8	9	10	11	12
*r*	0.912963	0.934806	0.898579	0.886813	0.926618	0.926618
SD	35.92265	27.35172	30.48291	33.92711	25.62175	25.62175
RMSE	349.7848	309.756	403.2535	399.4354	324.6582	324.6582
RE, %	−4.2897	−2.42926	3.505187	3.581921	−3.85078	−3.85078
AE, %	19.14858	14.42561	18.44123	19.64499	14.38603	14.38603

**Table 8:** Evaluation of ANN Pb model accuracy at various numbers of neurons in two hidden layers with Logsigmoid transfer function.

	7	8	9	10	11	12
*r*	0.904008	0.893182	0.920333	0.938538	0.900297	0.903822
SD	36.65264	39.09837	29.4667	24.77693	33.06996	31.92999
RMSE	360.6007	390.8199	326.3833	300.6916	369.6344	369.0974
RE, %	−8.8592	−5.23011	−7.32821	−1.70559	−5.38189	−7.06168
AE, %	19.72454	17.57162	17.77542	14.39224	18.6628	17.96397

**Table 9:** Characteristics of the ANN model for Pb prediction.

Parameter	Value
Layers number	4
Neurons (in the input layer)	4
Hidden layers number	2
Number of optimum neurons (in the hidden layer)	10
Training algorithm for the neural network	Levenberg−Marquardt
The transfer function of the hidden layer	Log sigmoid
The transfer function of the output layer	Pure linear

The following procedures can be used to provide the Pb model created with the ANN for each given data set:

**Step 1:** The subsequent expressions are employed to normalize the input parameters (Eqs. 2–5).

2 $$\:{\mathrm{R}}_{\mathrm{s}\mathrm{n}}=0.000474\mathrm{*}{\mathrm{R}}_{\mathrm{s}}-1.024645\:\:$$

3 $$\:{{\uprho\:}}_{\mathrm{g}\mathrm{n}}=1.818182\mathrm{*}{{\uprho\:}}_{\mathrm{g}}-2.090909$$

4 $$\:{{\uprho\:}}_{\mathrm{o}\mathrm{n}}=4.040404\mathrm{*}{{\uprho\:}}_{\mathrm{o}}-2.729293$$

5 $$\:{\mathrm{T}}_{\mathrm{n}}=0.006329\mathrm{*}\mathrm{T}-3.911392$$

**Step 2:** The subsequent equation (Eq. 6) is employed to compute the inputs for the initial hidden layer using the coefficients listed in supplementary material (Table S4).

6 $$\:\:three{\mathrm{S}}_{\mathrm{i}}=\sum\:_{\mathrm{i}=1}^{10}\left({\mathrm{W}}_{\mathrm{I},1}\times\:{\mathrm{R}}_{\mathrm{s}\mathrm{n}}+{\mathrm{W}}_{\mathrm{i},2}\times\:{{\uprho\:}}_{\mathrm{g}\mathrm{n}}+{\mathrm{W}}_{\mathrm{I},3}\times\:{{\uprho\:}}_{\mathrm{o}\mathrm{n}}+\right){\mathrm{W}}_{\mathrm{I},4}\times\:{\mathrm{T}}_{\mathrm{n}}+{\mathrm{b}}_{\mathrm{i}}$$

**Step 3:** The values of Si and the sigmoid function F are used to compute the outputs of the first hidden layer. The following expression (Eq. 7) using the coefficients listed in supplementary material (Table S5).

7 $$\:{X}_{i}=\frac{1}{1+\mathrm{e}\mathrm{x}\mathrm{p}\left({-\mathrm{S}}_{\mathrm{i}}\right)}$$

**Step 4:** The inputs and outputs of the second hidden layer are calculated using the coefficients listed in Table 1 as indicated in Eqs. 8–9.

8 $$\:{\mathrm{S}\mathrm{S}}_{\mathrm{i}}=\sum\:_{\mathrm{i}=1}^{10}\left({\mathrm{W}}_{\mathrm{i},\mathrm{j}}*{X}_{i}\right)+{\mathrm{b}}_{\mathrm{I},\mathrm{h}1}$$

9 $$\:{F}_{i}=X\left({\mathrm{S}\mathrm{S}}_{\mathrm{i}}\right)$$

**Step 5:** The Pb can be determined by using the following function (Eq. 10).

10 $$\:{P}_{b}=2419.5\left[\sum\:_{\mathrm{i}=1}^{10}\left({W}_{hi}{F}_{i}\right)+{\mathrm{b}}_{\mathrm{h}}\right]+2583.5$$

**Fig. 10:**
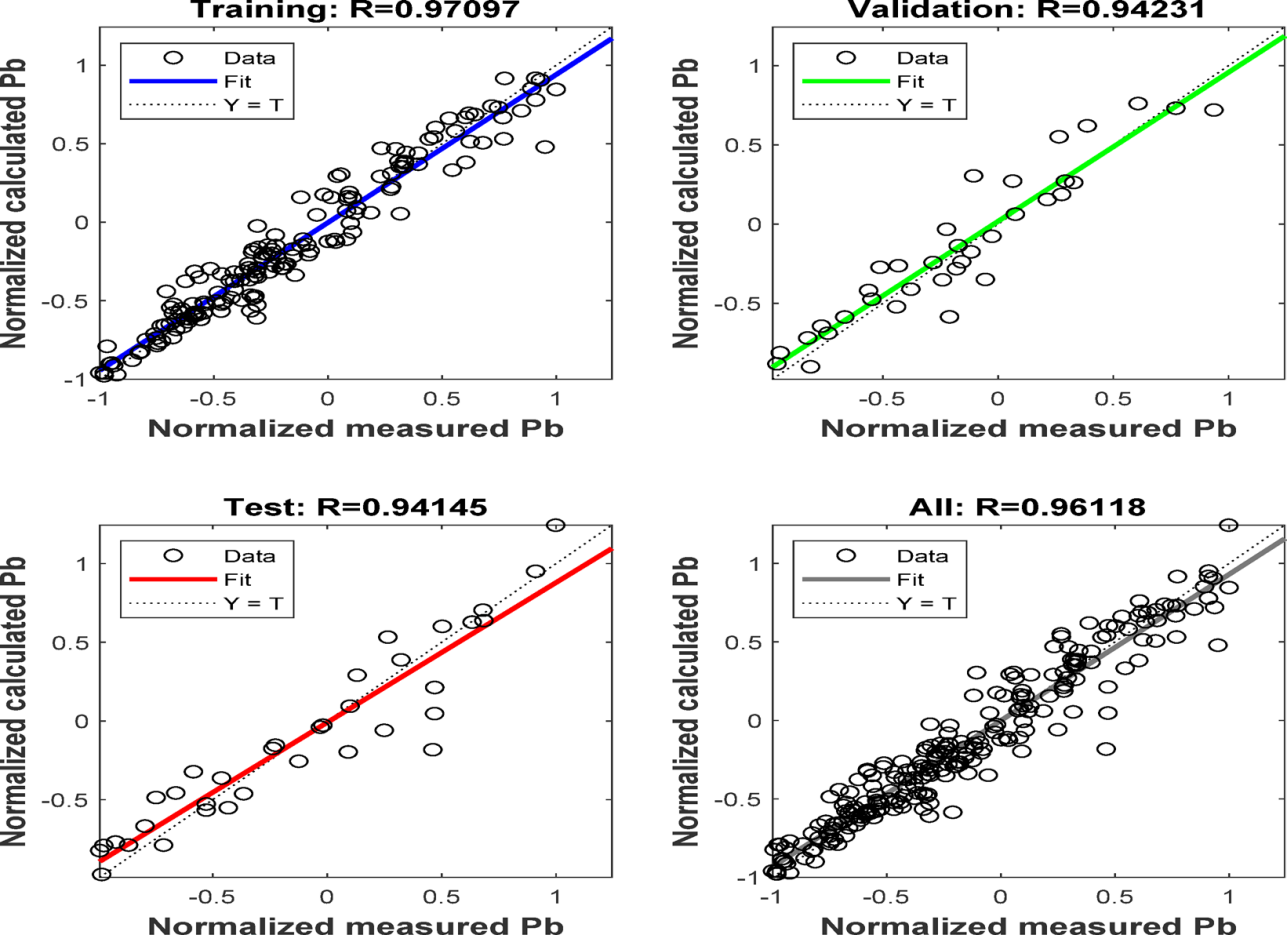
Cross plots of the ANN-Pb model.

The coefficients of Pb for the proposed model are presented in the supplementary material (Tables S4-S5). The number 1 denotes the first input parameter, solution GOR, the number 2 denotes the second input parameter, gas density, the number 3 denotes the third input parameter, oil density, and the number 4 denotes the fourth input parameter, reservoir temperature. Regression charts for the Pb model, as illustrated in Fig. 10, demonstrate the relationship between network outputs and targets for training, validation, testing, and all data points, with the coefficient of determination surpassing 0.99 across all plots.

## Conclusion

Accurate prediction of Pb is critical for optimizing reservoir and production engineering operations. This study introduced two new empirical correlations tailored for heavy and light oils, demonstrating superior predictive accuracy compared to existing models. By incorporating compositional analysis and leveraging advanced regression techniques, the proposed correlations achieved correlation coefficients of 99.08% for heavy oils and 96.49% for light oils. Additionally, an ANN model was successfully improved to estimate the Pb based on gas density, reservoir temperature, the solution Gas-Oil Ratio (GOR), and oil density. ANN models were developed to capture complex, non-linear relationships between input parameters, further enhancing prediction accuracy. The model comprises four layers: one input layer, two hidden layers with 10 neurons each, and one output layer. The ideal arrangement employed the log-sigmoid transfer function for the hidden layers and a pure-linear function for the output layer. The Levenberg-Marquardt algorithm was employed for training, yielding a substantial coefficient of determination (*r* > 0.99) and an AARE of 0.16. Normalization of input parameters and the incorporation of optimal weights and biases improved the model’s accuracy, as confirmed by regression plots for training, validation, and testing datasets. This robust ANN framework demonstrates its capability to accurately estimate bubble point pressure, highlighting its potential application in reservoir engineering workflows. Validation with independent datasets confirmed the reliability of these models, offering a significant advancement in Pb estimation for Egyptian oil reservoirs. These findings emphasize the value of region-specific models and advanced computational techniques in improving reservoir management and production optimization.

## Supplementary Information

Below is the link to the electronic supplementary material.

Supplementary Material 1

## Data Availability

The datasets used and/or analyzed during the current study available from the corresponding author on reasonable request.

## References

[1] Mansour, E. M., Aily, E. & Desouky, S. E. M. *Oil and Gas Wells* (IntechOpen, 2019).

[2] Mansour, E. M. & El Aily, M. Hydrocarbon simulation behavior of wet natural gas reservoirs. *Egypt. J. Chem.* **64**, 277–284 (2021).

[3] El-Hoshoudy, A. et al. New correlations for prediction of viscosity and density of Egyptian oil reservoirs. *Fuel* **112**, 277–282 (2013).

[4] El-Hoshoudy, A. PVT properties of black & crude oil: a review. *Pet. Coal* **61** (2019).

[5] Ahmed, T. *Equations of State and PVT Analysis* (Elsevier, 2013).

[6] Al-Marhoun, M. A. PVT correlations for Middle East crude oils. *J. Pet. Technol.* **40**, 650–666 (1988).

[7] Labedi, R. M. *SPE Latin America and Caribbean Petroleum Engineering Conference.* SPE-21164-MS (SPE).

[8] Dokla, M. E. & Osman, M. E. Correlation of PVT properties for UAE crudes. *SPE Form. Eval.* **7**, 41–46 (1992).

[9] Petrosky, G. Jr & Farshad, F. *SPE Annual Technical Conference and Exhibition?* SPE-26644-MS (SPE).

[10] De Ghetto, G., Paone, F. & Villa, M. *SPE Europec featured at EAGE Conference and Exhibition?* SPE-28904-MS (SPE).

[11] Hanafy, H., Macary, S., ElNady, Y., Bayomi, A. & El Batanony, M. *SPE Oklahoma City Oil and Gas Symposium/Production and Operations Symposium.* SPE-37439-MS (SPE).

[12] Akanni, O. & Nasr-El-Din, H. SPE Middle East Oil & Gas Show and Conference (SPE-172575-MS, 2015).

[13] Mehran, F., Movagharnejad, K. & Didanloo, A. A New Approach for Gas Lift Method Optimization for an Iranian Oilfield.

[14] Mazandarani, M. T. & Ebrahim, H. Modeling and simulation of industrial adiabatic fixed-bed reactor for the catalytic reforming of methane to syngas. *Eur Congr Chem. Eng*, 16–20 .

[15] Emara, R. A new oil formation volume factor correlation of Egyptian crude oils. *IARJSET*10.17148/IARJSET.2015.21124 (2015).

[16] Ikpabi, P. B. & Akinsete, O. O. Correlation for predicting bubble point pressure for 22.3 ≤ API ≥ 45 crude oils: A white-box machine learning approach. *Int. J Front. Eng. Technol. Res.* **3**, 015–027 (2022).

[17] Arwini, S. Improved Al-Marhoun correlation for bubble point pressure to fit Libyan crude oils. *Univ. Zawia J. Eng. Sci. Technol.* **2**, 14–22 (2024).

[18] El-hoshoudy, A., Ahmed, A., Gomaa, S. & Abdelhady, A. An artificial neural network model for predicting the hydrate formation temperature. *Arab. J. Sci. Eng.* **47**, 11599–11608 (2022).

[19] Gomaa, S. et al. Development of artificial neural network models to calculate the areal sweep efficiency for direct line, staggered line drive, five-spot, and nine-spot injection patterns. *Fuel* **317**, 123564 (2022).

[20] Gouda, A. et al. Development of an artificial neural network model for predicting the dew point pressure of retrograde gas condensate. *J. Pet. Sci. Eng.* **208**, 109284 (2022).

[21] Moghadam, J. N., Salahshoor, K. & Kharrat, R. Introducing a new method for predicting PVT properties of Iranian crude oils by applying artificial neural networks. *Pet. Sci. Technol.* **29**, 1066–1079 (2011).

[22] Al-Marhoun, M. & Osman, E. *Abu Dhabi international petroleum exhibition and conference.* SPE-78592-MS (SPE).

[23] El-Sebakhy, E. et al. *SPE Middle East oil and gas show and conference.* SPE-105698-MS (SPE).

[24] Moghadassi, A., Parvizian, F., Hosseini, S. & Fazlali, A. A new approach for estimation of PVT properties of pure gases based on artificial neural network model. *Braz. J. Chem. Eng.* **26**, 199–206 (2009).

[25] Numbere, O., Azuibuike, I. & Ikiensikimama, S. *SPE Nigeria Annual International Conference and Exhibition.* SPE-167586-MS (SPE).

[26] Baarimah, S. O., Gawish, A. A. & BinMerdhah, A. B. Artificial intelligence techniques for predicting the reservoir fluid properties of crude oil systems. *Int. Res. J. Eng. Technol. (IRJET)* **2**, 373–382 (2015).

[27] Adeeyo, Y. A. in *SPE Nigeria Annual International Conference and Exhibition.* SPE-184378-MS (SPE).

[28] Elkatatny, S. & Mahmoud, M. Development of a new correlation for bubble point pressure in oil reservoirs using artificial intelligent technique. *Arab. J. Sci. Eng.* **43**, 2491–2500 (2018).

[29] Ghorbani, H. et al. (2020).

[30] Alakbari, F. S., Mohyaldinn, M. E., Ayoub, M. A., Muhsan, A. S. & Hussein, I. A. A reservoir bubble point pressure prediction model using the Adaptive Neuro-Fuzzy Inference System (ANFIS) technique with trend analysis. *PLoS One* **17**, e0272790 (2022). 35951585 10.1371/journal.pone.0272790 PMC9371345

[31] Mansour, E. et al. Predicting PVT properties of Egyptian crude oils by a modified Soave–Redlich–Kowng equation of state. *Egypt. J. Pet.* **22**, 137–148 (2013).

[32] Gomaa, S., Emara, R., Mahmoud, O. & El-Hoshoudy, A. New correlations to calculate vertical sweep efficiency in oil reservoirs using nonlinear multiple regression and artificial neural network. *J. King Saud Univ. - Eng. Sci.* **34**, 368–375 (2022).

[33] Mansour, E. M. & Desouky, S. M. Improvement Wilson equation (K-values) of gas-liquid equilibrium for advancing estimating bubble point pressure. *Egypt. J. Chem.* **63**, 1941–1954 (2020).

[34] Mansour, E. et al. Modification proposed for SRK equation of state. *Oil Gas J. ***110** (2012).

[35] Mansour, E. (Cairo: Intech Open, 2020).

[36] Gomaa, S. et al. Machine learning prediction of methane, nitrogen, and natural gas mixture viscosities under normal and harsh conditions. *Sci. Rep.* **14**, 15155 (2024). 38956414 10.1038/s41598-024-64752-8 PMC11219757

[37] Arabloo, M. & Rafiee-Taghanaki, S. SVM modeling of the constant volume depletion (CVD) behavior of gas condensate reservoirs. *J. Nat. Gas Sci. Eng.* **21**, 1148–1155 (2014).

[38] Ghiasi, M. M., Shahdi, A., Barati, P. & Arabloo, M. Robust modeling approach for estimation of compressibility factor in retrograde gas condensate systems. *Ind. Eng. Chem. Res.* **53**, 12872–12887 (2014).

[39] ELAIL, M. Flashing losses emission evaluation from crude oil storage tanks. *Egypt. J. Chem.* **63**, 4457–4462 (2020).

[40] El Aily, M., Mansour, E., Desouky, S. & Helmi, M. Modeling viscosity of moderate and light dead oils in the presence of complex aromatic structure. *J. Pet. Sci. Eng.* **173**, 426–433 (2019).

[41] Elmabrouk, S., Zekri, A. & Shirif, E. *SPE Latin America and Caribbean Petroleum Engineering Conference.* SPE-137368-MS (SPE).

[42] Al-Marhoun, M. A. PVT correlations for Middle East crude oils. **40**, 650–666 (1988).

[43] Al-Shammasi, A. *SPE Middle East Oil and Gas Show and Conference.* SPE-53185-MS (SPE).

[44] Goldberg, S. I., Niemierko, A. & Turchin, A. *AMIA annual symposium proceedings.* 242 (American Medical Informatics Association).

[45] Ragab, A. & Mansour, E. Investigating the impact of PVT analysis errors on material balance calculations for oil reservoirs. *Pet. Coal* **62** (2020).

[46] Mansour, E., Desouky, S., Aily, E., Helmi, M. & M. & The effect of asphaltene content on predicting heavy dead oils viscosity: Experimental and modeling study. *Fuel* **212**, 405–411 (2018).

[47] El-Hoshoudy, A. Application of Artificial Intelligence and Federated Learning in Petroleum Processing. In *Artificial Intelligence Using Federated Learning* 134–155 (CRC Press, 2025).

