# Supplementary material for: Neural network and regression approaches for predicting bubble point pressure in oil reservoirs
Source: Sci Rep. 2026 Apr 30;16:13893. doi: 10.1038/s41598-026-49027-8 (PMC13133255; doi:10.1038/s41598-026-49027-8)
Supplement: Supplementary file 1 — Supplementary Material 1 [file 41598_2026_49027_MOESM1_ESM.docx]

Neural Network and Regression Approaches for Predicting Bubble Point Pressure in Oil Reservoirs

E.M. Mansour*1,2, Sayed Gomaa3 and A. N. El‑hoshoudy*1,2

^1^PVT lab, Production Department, Egyptian Petroleum Research Institute, 11727, Cairo, Egypt.

^2^ PVT service center, Egyptian Petroleum Research Institute, 11727, Cairo, Egypt.

^3^Mining and Petroleum Engineering Department, Faculty of Engineering, Al-Azhar University, Cairo, Egypt.

Corresponding author: E.M. Mansour, E-mail: [emanmansour84@yahoo.com](mailto:emanmansour84@yahoo.com).

A.N. El‑hoshoudy, E-mail: [azizchemist@yahoo.com](mailto:azizchemist@yahoo.com)

**Table S1: AI-based models**

| **Correlations** | **Year** | **Model Name** | **Data Number Samples** | **Data Source Samples** |
| --- | --- | --- | --- | --- |
| Marhoun and Osman | **(2002)** | Artificial Neural Networ( ANN) | 283 | Saudi Arabia |
| Sebakhy, Sheltami et al. | **(2007)** | Support Vector Machin (SVM) | --- | --- |
| Moghadassi, Parvizian et al. | **(2009)** | Artificial Neural Networ( ANN) | 150 | global  datasets |
| Moghadam, Salahshoor et al. | **(2011)** | Artificial Neural Networ( ANN) | 387 | Iranian |
| Numbere, Azuibuike et al | **(2013)** | Artificial Neural Networ( ANN) | 1,248 | Niger Delta |
| Baarimah, Gawish et al. | **(2015)** | Fuzzy Logic (FL) model | 760 | previously published  sources |
| Adeeyo | **(2016)** | Artificial Neural Networ( ANN) | 2114 | Nigeria |
| Elkatatny and Mahmoud | **(2018)** | - ANN - Support Vector Machin (SVM) . - Adaptive Neuro-Fuzzy   Inference System (ANFIS). | --- | --- |
| Ghorbani, Wood et al. | **(2020)** | CHPSO-ANFIS model | 565 | Iranian |
| Alakbari, Mohyaldinn et al. | **(2022)** | Adaptive Neuro-Fuzzy Inference System (ANFIS) | 700 | global  datasets |

**Table S2: Performance Evaluation Metrics**

| Metric | Formula |
| --- | --- |
| Relative Error (%), (E_r_) | $Er_{i}(\%)=\frac{\left\vert X_{i}-\hat{X}_{i}\right\vert}{X_{i}\times100}$ |
| Absolute Error (E_a_) | $E_{a}=\left\vert X_{i}-\hat{X}_{i}\right\vert$ |
| Standard Deviation of Errors (SD) | $\begin{matrix} SD=\sqrt{\frac{1}{N-1}\sum_{i=1}^{N} (e_{i}-\bar{e})^{2}} \\ e_{i}=X_{i}-\hat{X}_{i} \\ \bar{e}=\frac{1}{N}\sum e_{i} \end{matrix}$ |
| Correlation Coefficient (r) | $r=\frac{\sum_{i=1}^{n} (X_{i}-\bar{X})(\hat{X}_{i}-\bar{\hat{X}})}{\sqrt{\sum_{i=1}^{n} (X_{i}-\bar{X})^{2}\sum_{i=1}^{n} (\hat{X}_{i}-\bar{\hat{X}})^{2}}}$ |
| Maximum Absolute Error (E_max_) | $E_{\max}=\max(\left\vert X_{i}-\hat{X}_{i}\right\vert)$ |
| Minimum Absolute Error (E_min_) | $E_{\max}=\min(\left\vert X_{i}-\hat{X}_{i}\right\vert)$ |

**Table S3: Nomenclature**

| **Symbol / Abbreviation** | **Definition** |
| --- | --- |
| ANFIS | Neuro-Fuzzy Inference System |
| MSE | Mean Square Error |
| SVM | Support Vector Machine |
| PVT | Pressure-Volume-Temperature |
| AI | Artificial Intelligence |
| Pb | Bubble Point Pressure |
| Tres | Reservoir Temperature |
| GOR | Gas Oil Ratio |
| Rs | Solution Gas-Oil Ratio |
| API | API Gravity of Crude Oil |
| R | coefficient of determination |
| r | Correlation Coefficient |
| Er | Relative Error |
| Ea | Absolute Error |
| SD | Standard Deviation |
| Emax | Maximum Error |
| Emin | Minimum Error |
| ANN | Artificial Neural Network |
| GEP | Gene Expression Programming |
| ML | Machine Learning |

**Table S4: Weights and Biases between the first and second layers.**

| Neuron #, i | $W_{i,1}$ | $W_{i,2}$ | $W_{i,3}$ | $W_{i,4}$ | $b$ |
| --- | --- | --- | --- | --- | --- |
| 1 | 2.8308 | -3.3662 | 1.4531 | 1.8889 | -5.0071 |
| 2 | 2.1871 | 2.6195 | -2.5097 | -2.5707 | -3.8736 |
| 3 | -4.6821 | 2.4818 | 3.1968 | -2.6751 | 3.8262 |
| 4 | 2.6951 | -0.65632 | 3.3654 | -3.0333 | -1.8015 |
| 5 | 1.9232 | 2.1909 | 0.52921 | -4.1728 | -0.95873 |
| 6 | -4.4027 | -2.8642 | 2.5682 | 2.6361 | 0.048207 |
| 7 | -3.5172 | -0.10765 | 3.8775 | 1.4022 | -2.148 |
| 8 | -1.7343 | 2.7802 | -2.7364 | -4.5133 | 3.2186 |
| 9 | 2.7523 | 2.8248 | 2.6765 | 4.2924 | 4.6728 |
| 10 | 3.859 | 0.93709 | -1.1207 | -1.8502 | 4.7984 |

Table S5: Weights and Biases between the second and third layers and between the third and output layers.

| i | 1 | 2 | 3 | 4 | 5 | 6 | 7 | 8 | 9 | 10 |
| --- | --- | --- | --- | --- | --- | --- | --- | --- | --- | --- |
| Wi,1 | -0.42731 | -0.63164 | 2.3089 | 2.5682 | -1.9944 | -0.11862 | -0.49082 | 1.2028 | 1.7439 | 2.4851 |
| Wi,2 | -1.8687 | 1.1852 | 1.3488 | -2.8698 | -2.2433 | 0.1382 | 3.8049 | -1.3703 | 0.71289 | -2.7147 |
| Wi,3 | 1.928 | -2.0381 | 0.073479 | 1.6873 | 1.231 | 4.2557 | 1.1283 | -3.7083 | -2.7927 | -2.178 |
| Wi,4 | 2.6777 | -0.72778 | 2.7325 | -2.0818 | 2.7291 | -1.6233 | -2.4087 | -2.5852 | 1.039 | -0.2743 |
| Wi,5 | -1.3465 | 2.2112 | 0.74045 | 0.42968 | 2.5452 | -1.7916 | 1.9864 | 2.1065 | -0.95961 | 0.33488 |
| Wi,6 | -3.6062 | -3.1009 | 0.28922 | 2.2089 | 1.6746 | -4.096 | 0.36163 | -0.9163 | -4.1437 | -1.4674 |
| Wi,7 | -3.1728 | 1.7067 | -1.6219 | 0.32122 | -3.8336 | -0.26826 | -2.4884 | 1.6372 | 1.9108 | 3.96 |
| Wi,8 | -0.63816 | -1.7883 | -2.3544 | 3.7146 | -2.1304 | 2.7652 | 0.61527 | 4.5533 | -3.6194 | -1.8028 |
| Wi,9 | -2.881 | 3.7697 | -4.2981 | 1.9706 | 2.4068 | 1.5492 | -3.3453 | -2.9239 | -2.3886 | 2.2355 |
| Wi,10 | -0.06124 | 3.9465 | -2.7665 | -2.3487 | -2.6619 | -3.3344 | 3.3369 | 0.72772 | 0.002801 | -4.274 |
| bi1h1 | 8.3012 | 1.6303 | -0.12567 | -4.3506 | -0.83872 | -0.10379 | -1.5561 | 0.86025 | 6.7826 | 4.8782 |
| Wih2 | 0.56861 | 2.673 | -0.36989 | 1.5957 | -2.7304 | -3.3721 | -1.5831 | -1.4475 | 0.83433 | -1.4418 |
| bh2 | -0.12839 |  |  |  |  |  |  |  |  |  |

**Data Availability:** The datasets used and/or analyzed during the current study available from the corresponding author on reasonable request.

**Funding Source:** No Funds were extended for this work.

**Conflict of interest:** The author declares no conflict of interest
